# Specimen‐driven intraoperative assessment of resection margins should be standard of care for oral cancer patients

**DOI:** 10.1111/odi.13619

**Published:** 2020-09-13

**Authors:** Yassine Aaboubout, Ivo ten Hove, Roeland W. H. Smits, Jose A. Hardillo, Gerwin J. Puppels, Senada Koljenovic

**Affiliations:** ^1^ Depatment of Pathology Erasmus MC University Medical Center Rotterdam Rotterdam The Netherlands; ^2^ Department of Otorhinolaryngology and Head and Neck Surgery Erasmus MC University Medical Center Rotterdam Rotterdam The Netherlands; ^3^ Department of Oral and Maxillofacial Surgery Erasmus MC University Medical Center Rotterdam Rotterdam The Netherlands; ^4^ Department of Oral and Maxillofacial Surgery LUMC Leiden University Medical Center Rotterdam The Netherlands; ^5^ Department of Dermatology Erasmus MC University Medical Center Rotterdam Rotterdam The Netherlands

**Keywords:** head and neck cancer, intraoperative assessment, oral cancer, resection margin, squamous cell carcinoma, surgery

## Abstract

With an incidence of 350.000 new cases per year, cancer of the oral cavity ranks among the 10 most common solid organ cancers. Most of these cancers are squamous cell carcinomas. Five‐year survival is about 50%. It has been shown that clear resection margins (>5 mm healthy tissue surrounding the resected tumor) have a significant positive effect on locoregional control and survival. It is not uncommon that the resection margins of oral tumors are inadequate. However, when providing the surgeon with intraoperative feedback on the resection margin status, it is expected that obtaining adequate resection margins is improved. In this respect, it has been shown that specimen‐driven intraoperative assessment of resection margins is superior to defect‐driven intraoperative assessment of resection margins. In this concise report, it is described how a specimen‐driven approach can increase the rate of adequate resections of oral cavity squamous cell carcinoma as well as that it is discussed how intraoperative assessment can be further improved with regard to the surgical treatment of oral cavity squamous cell carcinoma.

## 1 INTRODUCTION

Each year about 350.000 patients are diagnosed with cancer in the oral cavity worldwide, the vast majority of which are squamous cell carcinoma (90% of the cases) (Bray et al., 2018). Oral cavity squamous cell carcinoma (OCSCC) ranks among the ten most common solid organ cancers. The 5‐year survival of OCSCC patients is about 50%, with little improvement over the last decades (van der Ploeg, Datema, Baatenburg de Jong, & Steyerberg, 2014).

Surgery is the mainstay of treatment for OCSCC and aims for complete resection of the tumor with adequate margins, while sparing healthy tissue as much as possible (Shah & Gil, 2009).

The most widely accepted definition of margins in oral cancer surgery is that of The Royal College of Pathologists (Meier, Oliver, & Varvares, 2005). A clear (or adequate) margin means a distance of more than 5 mm from resection surface to the tumor border, a distance of 1–5 mm is called a close margin. A distance of <1 mm is called a positive margin (Helliwell & Woolgar, 2013).

Of all oncological prognostic factors (i.e., patient and tumor characteristics), the surgeon and pathologist can only influence the resection margins. Adequate resection margins in OCSCC lead to higher survival and a marked reduction in local recurrence (Smits et al., 2016; Varvares, Poti, Kenyon, Christopher, & Walker, 2015). Inadequate resection margins result in the need for adjuvant therapy in the form of postoperative (chemo‐) radiotherapy. Adjuvant therapy brings an additional burden for the patient, which in the vast majority of cases results in increased morbidity and reduced quality of life (Lin, 2018).

There is a debate in the literature about the margin definition. Although there is evidence that margins of >5 mm improve patient outcome (e.g., local control, disease‐free survival, overall survival) and that there should be agreement on 5 mm margin as a clear margin (Meier et al., 2005; Smits et al., 2016; Varvares et al., 2015), several studies found that margins of <5 mm are sufficient, especially for early‐stage OCSCC. Nason, Binahmed, Pathak, Abdoh, and Sándor (2009) unequivocally stated that survival improves with each additional millimeter of clear surgical margin and proposes a minimum margin of 3 mm to be considered an adequate resection. Zanoni et al. (2017) showed that for tongue cancer, resection margins between 2.2 and 5 mm show no greater risk of local recurrence, than margins >5 mm. Jang et al. (2017) reported little or no effect of resection margin status on local recurrence, but only for small (<3 mm diameter) T1 tumors, as did Barry et al. (2014) for T1/T2 tumors. Dik et al. (2014) concluded that a margin of 3 mm with ≤2 other adverse histological features is as safe as a margin of 5 mm in relation to local recurrence. Another recent study showed that only a margin of <1 mm was associated with an increased risk of local recurrence (Buchakjian et al., 2018). However, the evidence put forward to decide what is an adequate margin is still very fragmented. Until sufficient evidence is accumulated in a meta‐analysis on the basis of which a new consensus can be reached, a margin >5 mm should be pursued.

A separate discussion concerns the recommended adjuvant therapy in connection with margin status. Many centers regard a positive margin to be an absolute indication for adjuvant treatment. There is no consensus on when to indicate adjuvant therapy in case of a close margin. However, many authors do not regard close margin (<5 mm) as adequate, but do not recommend postoperative radiotherapy for OCSCC patients if a close margin is the only adverse tumor feature (i.e., without perineural invasion and infiltrative growth pattern) (Ch’Ng et al., 2013). Dik et al. (2014) showed that there was no evidence of benefit for any local adjuvant therapy in case of a margin of 3 mm with only one or two more adverse histological features. They compared the impact of re‐resection, postoperative radiotherapy, and watchful waiting.

Working in the complex oral anatomy and having to rely solely on visual inspection, palpation, and preoperative imaging, the surgeon is caught between the goals of achieving an adequate tumor resection and safeguarding satisfactory remaining function and acceptable physical appearance.

Recent studies have shown that an adequate tumor resection is often only achieved in a minority (15%–26%) of cases (Dik et al., 2014; Smits et al., 2016; Varvares et al., 2015).

However, there is a wide range of adequate resection margins reported in the literature, varying from 35% to 70% (Smits et al., 2016). Surprisingly, clinical outcomes in terms of overall survival and recurrence seemed comparable among the centers, irrespective of the reported rate of adequate resections. This variation in results is caused by a lack of unanimous agreement on resection margins and differences in surgicopathological approaches. This prevents a genuine comparison of the results between the centers.

Clearly, the hands and eyes of the surgeon cannot warrant an adequate resection. Moreover, the definitive margin status, as determined during the final pathology, follows only several days after the operation. If at that point an inadequate margin is encountered, a second operation is not an option, nor effective, because an accurate relocation of the site of an inadequate margin is impossible in most cases. Therefore, there is a need for the introduction of techniques to improve getting adequate surgical margins.

## 2 HOW TO ACHIEVE “FIRST TIME RIGHT” SURGERY?

It is evident that for optimal control of resection margin, the surgeon needs additional information. Intraoperative assessment of resection margins (IOARM) can provide such valuable information, enabling additional tissue resection when needed to turn an otherwise inadequate tumor resection into an adequate operation. Two methods of IOARM can be distinguished: the traditional defect‐driven IOARM based on frozen sections and the recently recommended specimen‐driven assessment.

### 2.1 Defect‐Driven IOARM

For defect‐driven intraoperative assessment, the surgeon takes tissue samples from the wound bed for frozen section histopathologic analysis. Of all surgical disciplines, intraoperative assessment of the resection margins based on the frozen section procedure is most often performed for head and neck cancers (McIntosh, Harada, Drwiega, Brandwein‐Gensler, & Gordetsky, 2015).

Although the frozen section analysis is a well‐known procedure available in many centers, studies have reported that it has no impact on regional control or an improvement in survival in OCSCC patients (Abbas, Ikram, Tariq, Raheem, & Saeed, 2017; Buchakjian et al., 2018; Buchakjian, Tasche, Robinson, Pagedar, & Sperry, 2016; Mair et al., 2017; Pathak et al., 2009; Varvares et al., 2015).

Frozen section analysis during defect‐driven IOARM has a high accuracy in classification of the tissue samples, but is poorly predictive of the final margin status. The obvious reason is that the method is time‐consuming and laborious, so that relatively few tissue samples can be analyzed intraoperatively. Hence, the method is fraught with sampling error. Recent large cohort studies showed no benefit with respect to local recurrence or survival, when a re‐resection was performed because of a positive frozen section margin based on defect‐driven intraoperative assessment (Buchakjian et al., 2016,2018). This is caused by the well‐known difficulty of relocation of the exact location of the frozen section tissue sample in the wound bed. Relocation is particularly difficult in the head and neck region, and therefore, an optimal additional resection is not always achieved (Gokavarapu et al., 2015; Kerawala & Ong, 2001; Magliocca, 2017; Maxwell et al., 2015; Williams, 2016).

Thus, the defect‐driven frozen section procedure is presumed to be insufficient for decision making regarding the need for additional resection to achieve “first time right” surgery.

### 2.2 Specimen‐driven IOARM

A 2005 survey reported that over 90% of surgeons performed a defect‐driven frozen section analysis and only 14%–24% performed a specimen‐driven margin assessment during OCSCC surgery (Meier et al., 2005).

Since that time, there is growing evidence that specimen‐driven IOARM is superior to defect‐driven assessment (Amit et al., 2016; Hinni, Zarka, & Hoxworth, 2013; Kain et al., 2020; Maxwell et al., 2015; Varvares et al., 2015). A recent study showed that specimen‐driven IOARM by macroscopic examination and measurement of margins is as accurate as specimen‐driven IOARM accompanied by sampling of tissue for microscopic evaluation of frozen sections (Mair et al., 2017).

**Figure 1:**
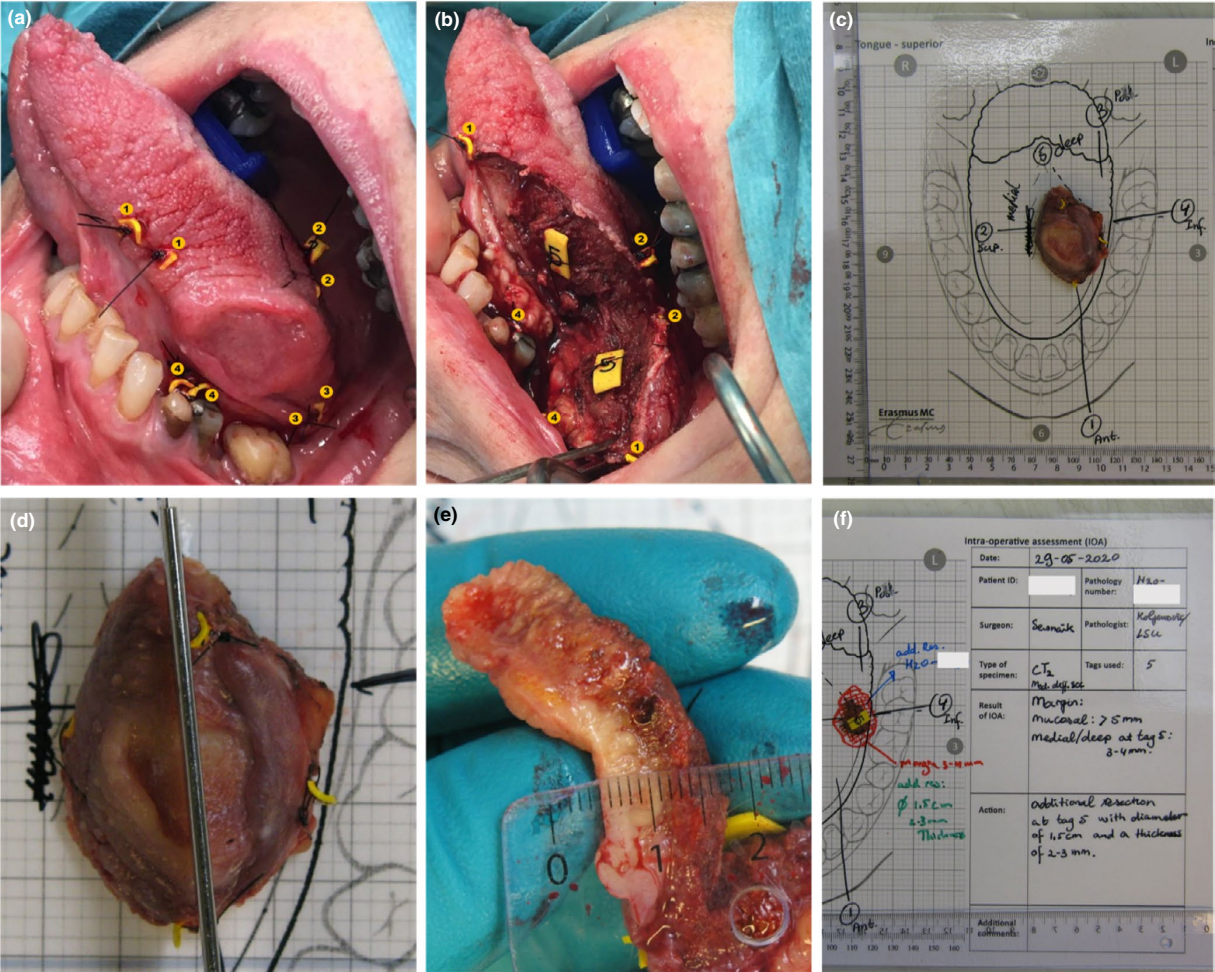
Illustration of the IOARM procedure including the relocation method by parallel tagging. (a) The surgeon attaches numbered tags in a pair‐wise manner on both sides of the resection line, superficial and deep during the resection. (b) After the tumor resection has been completed, one numbered tag of each pair is attached to the specimen and the other tag remains in the wound bed. (c) Anatomical template of the tongue with the specimen, patient information, and the annotated tags. These templates have been designed to facilitate the preservation of anatomical orientation of the specimen during the IOARM. (d) The pathologist and surgeon inspect and palpate the specimen for suspicious areas (i.e., areas where margin might be less than ≤ 5 mm). If a suspicious area is found, the pathologist makes one or more parallel incisions perpendicular to the resection surface (with a mutual distance of 5–6 mm). This enables the visualization and measurement of the margin. (e) Measuring the margin with a ruler. If an inadequate margin is detected, its location is indicated based on the numbered tags. Advice is given for an additional resection in the indicated area, including the thickness. (f) Result of IOARM (e.g., *at the location of tag nr. 5, the margin is 3–4 mm*) is recorded at the template, together with the recommendation for additional resection (e.g., *area of tissue enclosing tag 5, with a diameter of 1.5 cm and the thickness 3–4 mm*)

In 2017, the American Joint Committee on Cancer (AJCC) has recommended specimen‐driven intraoperative assessment as a standard of care (Amin et al., 2017). At our institute, we have implemented a comprehensive specimen‐driven IOARM since 2013. The method has become our standard of care in 2015 (Figure 1). A pathologist and the surgeon inspect the resection specimen macroscopically (by visual inspection, palpation, and by making incisions in the specimen perpendicular to the resection plane) and when necessary microscopically (by sampling tissue for frozen section analysis, from the suspicious areas if the location of the tumor border is not clear by macroscopic inspection).

The specimen‐driven IOARM procedure is accompanied by a simple method for the relocation of inadequate margins in the wound bed, that have been identified on the resection specimen, to enable confident additional resection. The relocation method is described in detail by van Lanschot et al. (2019).

Preferably, the entire IOARM process, including the conclusion and the recommendation for additional resection, is recorded (including photographs) and stored in the patient file. This information can then be used during the final pathologic assessment and multi‐disciplinary consultations.

Although specimen‐driven IOARM has led to a significant improvement in obtaining adequate OCSCC surgical margins, which underlines the necessity of intraoperative feedback to the surgeon, the level of its wide implementation still leaves a lot to wish for.

The main concerns to perform specimen‐driven IOARM include the fact that grossing fresh tissue is counter‐intuitive to pathologists as wells as that grossing fresh tissue might deteriorate the anatomical orientation and the shape or size of the specimen. These obstacles potentially can affect the final, postoperative pathologic assessment (Pangare et al., 2017; Umstattd, Mills, Critchlow, Renner, & Zitsch, 2017). Another concern is the assumption that a specimen‐driven IOARM might be more time‐consuming than defect‐driven IOARM, because of the distance between the operating room and department of pathology. Finally, it is not realistic to expect that this approach can be commonly adopted because a dedicated team of head and neck surgeons and pathologists is not available in every center.

## 3 FUTURE

Specimen‐driven IOARM works, but it is important to be open for innovative modalities with the goals to further improve its accuracy and to enable more widespread implementation. For example, technology is needed that will enable objective inspection of the entire resection surface. Raman spectroscopy is among the most promising optical techniques to fill this gap. Raman spectroscopy is an optical technique that does not require sample preparation. This technique provides real‐time information about the molecular composition of the tissue. Earlier studies have shown that Raman spectroscopy discriminates between OCSCC and healthy tissue, with a sensitivity of 99% and a specificity of 92% (Barroso et al., 2015,2016).

**Figure 2:**
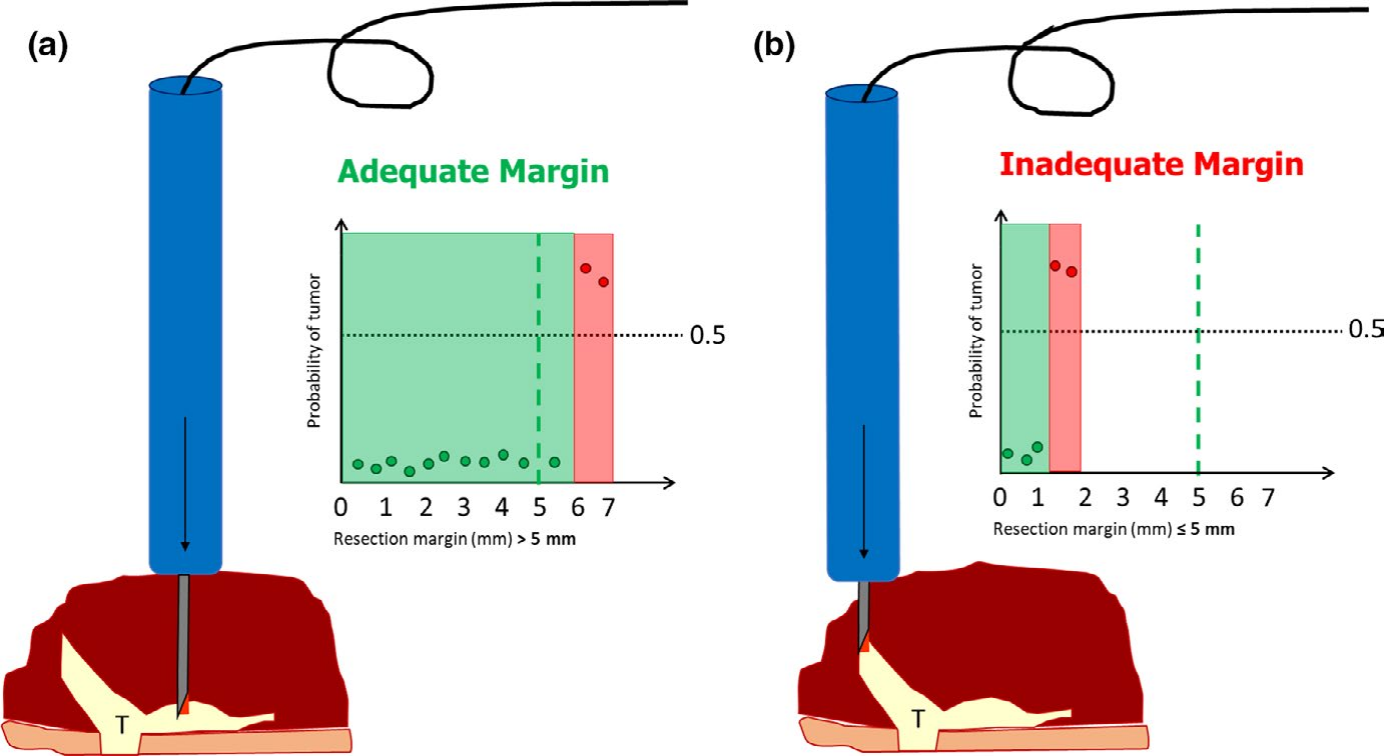
Illustration of specimen‐driven IOARM based on Raman spectroscopy. The fiber‐optic needle determines the resection margin as a distance between the resection surface and tumor border, given in millimeters. The fiber‐optic needle is driven into the specimen, from the resection surface toward the tumor border. Raman spectra are collected along the insertion path at each 0.5 mm of depth. In the graphs, each measurement is presented as a dot; the *x*‐axis shows the measured resection margin in millimeters, and the *y*‐axis shows the probability of individual measurements to be classified as tumor or not. (a) Example of adequate margin (6 mm) between tags 1 and 2, no additional resection is needed. (b) Example of inadequate margin (1.5 mm) between tags 0 and 1, an additional resection is needed

Currently, Raman spectroscopy is implemented in a prototype instrument employing a fiber‐optic needle probe (Figure 2). This fiber‐optic needle is driven into the specimen, from the resection surface toward the tumor. Based on the Raman spectra collected along the insertion path, it is determined whether the needle tip is in healthy or tumor tissue. This principle is used to measure the resection margin (i.e., distance between the resection surface and the tumor border, given in millimeters). This takes a few seconds per measurement and enables objective measurement of resection margins without the need for grossing of the specimen.

In addition, to be used for soft tissue intraoperative assessment, Raman spectroscopy can also be used to assess osseous resection margins in the OCSCC patients treated with bone resection (Barroso et al., 2018). When shown to be feasible and reliable, this Raman spectroscopic approach could solve the persisting problem of the lack of IOARM for bone resection margins (for both segmental and marginal bone resections) (Nieberler et al., 2014; Smits et al., 2018).

## 4 CONCLUSION

Radical tumor resection is the goal of surgery since ancient times, when Galen recommended that the whole tumor with its all “roots” should be removed (Papavramidou, Papavramidis, & Demetriou, 2010). Unfortunately, after almost two millennia this goal is still not achieved for many patients. The importance of adequate tumor resection cannot be overemphasized, and specimen‐driven intraoperative assessment of resection margins is crucial to this. In addition to the upcoming specimen‐driven IOARM approach, new technology is needed to further improve its accuracy and to enable its widespread implementation.

The literature on IOARM is clear in its verdict that a specimen‐driven approach is superior to defect‐driven IOARM in guiding surgeon and pathologist toward adequate resection.

## CONFLICT OF INTEREST

None to declare.

## AUTHOR CONTRIBUTION

**Yassine Aaboubout:** Conceptualization; Data curation; Investigation; Methodology; Project administration; Resources; Software; Visualization; Writing‐original draft; Writing‐review & editing. **Ivo ten Hove:** Data curation; Investigation; Methodology; Resources; Software; Validation; Visualization; Writing‐original draft; Writing‐review & editing. **Roeland W. H. Smits:** Investigation; Methodology; Resources; Writing‐review & editing. **Jose A. Hardillo:** Investigation; Methodology; Resources; Writing‐review & editing. **Gerwin J. Puppels:** Conceptualization; Data curation; Funding acquisition; Investigation; Methodology; Project administration; Resources; Software; Supervision; Validation; Visualization; Writing‐original draft; Writing‐review & editing. **Senada Koljenovic:** Conceptualization; Formal analysis; Funding acquisition; Investigation; Methodology; Project administration; Resources; Software; Supervision; Validation; Visualization; Writing‐original draft; Writing‐review & editing.

### PEER REVIEW

The peer review history for this article is available at https://publons.com/publon/10.1111/odi.13619.
